# Sensor Fusion Algorithm Using a Model-Based Kalman Filter for the Position and Attitude Estimation of Precision Aerial Delivery Systems

**DOI:** 10.3390/s20185227

**Published:** 2020-09-13

**Authors:** Raul A. Garcia-Huerta, Luis E. González-Jiménez, Ivan E. Villalon-Turrubiates

**Affiliations:** Instituto Tecnológico y de Estudios Superiores de Occidente (ITESO), 45604 Tlaquepaque, Jalisco, Mexico; luisgonzalez@iteso.mx (L.E.G.-J.); villalon@iteso.mx (I.E.V.-T.)

**Keywords:** flight dynamics, Kalman filter, linear model, precision aerial delivery system (PADS), sensor fusion, unmanned aerial vehicle (UAV)

## Abstract

In this research, we focus on the use of Unmanned Aerial Vehicles (UAVs) for the delivery of payloads and navigation towards safe-landing zones, specifically on the modeling of flight dynamics of lightweight vehicles denoted Precision Aerial Delivery Systems (PADSs). While a wide range of nonlinear models has been developed and tested on high-end applications considering various degrees of freedom (DOF), linear models suitable for low-cost applications have not been explored thoroughly. In this study, we propose and compare two linear models, a linearized version of a 6-DOF model specifically developed for micro-lightweight systems, and an alternative model based on a double integrator. Both linear models are implemented with a sensor fusion algorithm using a Kalman filter to estimate the position and attitude of PADSs, and their performance is compared to a nonlinear 6-DOF model. Simulation results demonstrate that both models, when incorporated into a Kalman filter estimation scheme, can determine the flight dynamics of PADSs during smooth flights. While it is validated that the double integrator model can adequately operate under the proposed estimation scheme for up to small acceleration changes, the linearized model proves to be capable of reproducing the nonlinear model characteristics even during moderately steep turns.

## 1. Introduction

The use of Unmanned Aerial Vehicles (UAVs) has spread to numerous disciplines and will continue to expand in the foreseeable future, as more affordable sensors, actuators, and processing units become available, together with the advances in their legislation around the globe. Nowadays, UAVs are used in several applications in the scientific, civil, and commercial fields; for instance, in precision agriculture, weather monitoring, search and rescue, mining, remote sensing, and delivery of goods [1,2].

In this research, we focus on the use of UAVs for the delivery of payloads. A large number of applications make use of UAVs to deliver food, merchandise, medical supplies, or rescue equipment, among others. When natural disasters occur, such as earthquakes, landslides, blizzards, wildfires, or floods, affecting the terrestrial infrastructure, UAVs can promptly deliver emergency supplies. Additionally, UAVs provide a solution in situations where the rugged topography of a region inhibits fast transportation. For example, the company Zipline is delivering daily blood and blood products to health centers in the regions around the countries of Rwanda and Ghana, in Africa [3]. Given the coronavirus disease 2019 (COVID-19) pandemic, Zipline is also distributing medical supplies and personal protective equipment, and recently pursuing the possibility to provide COVID-19 supplies in the USA [4].

**Figure 1:**
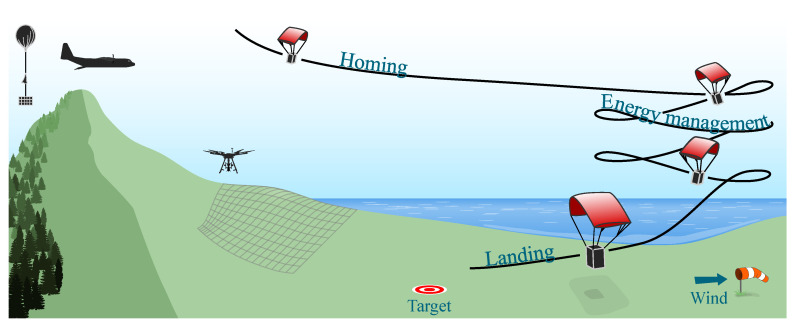
Typical PADS flight profile.

Several applications of UAVs rely on vehicles instrumented with expensive and delicate sensors. For instance, in remote sensing scenarios, it is common the use of Light Detection and Ranging (LiDAR); optical, multispectral, hyperspectral, and thermal cameras; radar; global navigation satellite system (GNSS) receivers; and processing units [5]. In the case of a vehicle malfunction, these instruments could be damaged given a collision with the ground, or lost in the event of loss of flight control or communication, causing landing in an unknown or unreachable location. In these situations, a safe-landing feature is most needed to recover the vehicle and its equipment. Precision Aerial Delivery Systems (PADSs) make use of guidance, navigation, and control, to maneuver parafoil-payload systems towards safe-landing zones, increasing the chances of successfully delivering payloads on target, and diminishing the possible damage while landing. A typical descent flight of a PADS is illustrated in Figure 1. The trajectory is composed of three segments: an initial flight towards the intended landing point (homing); a lemniscate (figure-eight) or circular pattern while losing altitude (energy management); and finally, a narrow turn to reach the target facing into the wind (landing). In this illustration three vehicles are depicted to represent different application scenarios, for example, a cargo aircraft for the delivery of goods, or as a recovery system for a weather balloon or a multicopter.

Design, modeling, and characterization of PADSs have been taking place since the beginning of the 1970s. Static and dynamic longitudinal stability was first studied by Goodrick, together with the early development of the equations of motion governing parafoil-payload systems, considering 3- and 6-degrees of freedom (DOF) [6,7], and the characterization of a 150 kg test payload. In the following years, the Small Autonomous Parafoil Landing Experiment (ALEX I and II), described in detail by Jann [8,9], set a milestone on modeling, validation, and verification of PADSs using 3- and 4-DOF, employing a ram-air parachute with a payload of 100 kg.

Different models have been developed for a varying range of DOF, either considering the parafoil-payload system as a rigid body or allowing relative motion between their components [10,11,12,13]. Comparisons between models happen to be a complex task, since different considerations, payload masses, parafoil aerodynamic properties, and general assumptions are used by different authors. To be able to compare the effectiveness between models ranging from 6- to 9-DOF, Gorman and Slegers [14,15] developed a parafoil model using the same aerodynamic properties for each scenario, realizing different DOF by modifying the kinematics constraints between the parafoil and the payload, under the same control inputs and initial conditions. The 6-DOF model represents the three inertial position components of the joint connecting the parafoil and the payload, as well as three Euler orientation angles. The 7-, 8-, and 9-DOF models incorporate extra Euler orientation angles for the payload, depending on the connection constraints. The 7-DOF model allows for yaw relative motion, the 8-DOF model allows yaw and pitch, whereas the 9-DOF model allows yaw, pitch, and roll relative motion between the parafoil and the payload. By contrasting experimental data with the models, Gorman and Slegers were able to conclude that the 6-DOF trajectory differed from the 7-, 8-, and 9-DOF trajectories mainly because of the great yaw relative motion (parafoil-payload twist). Since the relative roll and pitch motion is negligible in comparison to the relative yaw motion, the 7-DOF represents the model with the minimum DOF that captures the most significant flight dynamics, while the 6-DOF successfully characterizes the parafoil-payload system as a rigid body, presenting lower complexity than the rest of the proposed models. Since the PADS analyzed in this investigation is considered a rigid body, a 6-DOF model is adopted.

Due to the versatility of these delivery systems, and their possible applications in military scenarios, different sectors of the US Army teamed to form the Joint Precision Airdrop System (JPADS), categorizing their systems by weight as micro-light (4.5 kg to 68 kg), ultra-light (113 kg to 318 kg), extra-light (318 kg to 998 kg), light (2268 kg to 4536 kg) and medium (6804 kg to 19,051 kg) [16,17]. Initially, one of the JPADS requirements was to achieve $13.22 USD per kilogram of payload, a goal only met for the heavy categories, but far from being met for the light categories (micro-, ultra-, and extra-light) [18]. Making more affordable systems has been a priority, even at the expense of reducing their delivery precision. The Affordable Guided Airdrop System (AGAS), formed in 1999 [19], has the goal to minimize as much as possible the cost of delivery systems, obtaining the best achievable accuracy within a threshold of 100 m circular error probability (CEP). The AGAS initiative has been focusing on the extra-light and lightweight ranges, where mainly 900 kg payload implementations have been tested using circular parachutes, achieving 211 m CEP when 12 hour-old wind forecast information was used, and 38 m CEP when near real-time wind profiles were used [20]. Lighter systems have been able to achieve up to 10 m in specific cases [18]. Reduction in the actual landing distance to the intended point of impact, which translates to small CEP, has been achieved by the development of optimal control and guidance [21,22], strongly dependent on high-quality information about the wind profiles during the entire flight, from the beginning of the descent up to touchdown, and the capability to account for variable winds [23]. Nevertheless, achieving such high precision comes with a price, requiring computational power and elevated costs on sensors, actuators, and parachutes.

Although the 5 to 19,000 kg weight-range has been widely analyzed and tested, delivery systems for lighter applications have not been thoroughly explored, especially for low-cost solutions. Lightweight applications of PADSs face the challenge of relying on light hardware to perform guidance, navigation, and control. Current parafoil-payload systems make use of high-end processing units, capable of handling sophisticated real-time implementations of highly nonlinear dynamical models to estimate their attitude and position to operate their guidance system. If low-cost miniaturized versions of these delivery systems are to be explored, simplified models that capture their flight dynamics need to be developed. Additionally, estimation schemes for their position and attitude need to be taken into account, especially for further guidance and control implementations on vehicles with limited sensors, actuators, and processing capabilities.

The state estimation process based on the fusion of dynamic models and measurements obtained from different sensors is commonly and efficiently performed through a model-based Kalman filter. Whether a total state-space formulation of a Kalman filter is used; an error state-space, either with a feedforward or feedback implementation; an extended Kalman filter; an unscented Kalman filter; or any of modern developed variants, a Kalman filter is one of the most used algorithms for sensor fusion, particularly in navigation applications [24,25,26,27]. The type of Kalman filter implementation strongly depends on the intended application, the dynamics of the process to estimate, the required accuracy, and the computational capabilities at disposal.

According to a study performed by Zhang et al. [28], in which the performance of a Kalman filter, an extended Kalman filter, an unscented Kalman filter, and variations of these types of filters were compared for inertial navigation systems, the best accuracy is obtained by the unscented Kalman filter for their experiments. However, the unscented Kalman filter is the algorithm that demands the most computational effort among their comparison. On the other hand, the computational time required for the Kalman filter (not extended nor unscented) showed to be the lowest, 3 to 10 times smaller than the extended or unscented Kalman filter implementation, at the cost of having six to eight times smaller accuracy in the estimates.

Following the current development needs of PADSs, particularly for the micro-lightweight category, as well as the goals established by the AGAS program, in which the improvements of low-cost systems are prioritized even at the expense of decreasing the landing accuracy, a total state-space formulation of a Kalman filter is adopted for the estimation scheme proposed in this investigation. This type of implementation enables the use of low-cost, small, and lightweight sensors and processing units, requiring a linear model that represents the flight dynamics of the system.

This research presents the development, implementation, and comparison of a sensor fusion algorithm and estimation scheme for the position and attitude of PADSs, employing a Kalman filter based on two proposed 6-DOF dynamic models, suitable for lightweight low-cost applications. Section 2 details the proposed estimation scheme. The dynamic models that characterize the flight of the parafoil-payload system are presented in Section 3, firstly introducing a nonlinear 6-DOF dynamic model, and then the development of two linear alternatives: a linearized version of the nonlinear 6-DOF model, and a double integrator model. The required sensors and their characteristics suitable to perform the position and attitude estimation are presented in Section 4, whereas Section 5 presents the Kalman filter algorithm that incorporates the dynamic models and measurements from different sensors. The performance of the proposed estimation scheme is evaluated through simulations in Section 6, followed by the discussion of the results in Section 7. Finally, the conclusions of the proposed estimation scheme are outlined and the considered future work is depicted.

## 2. Proposed Estimation Scheme

**Figure 2:**
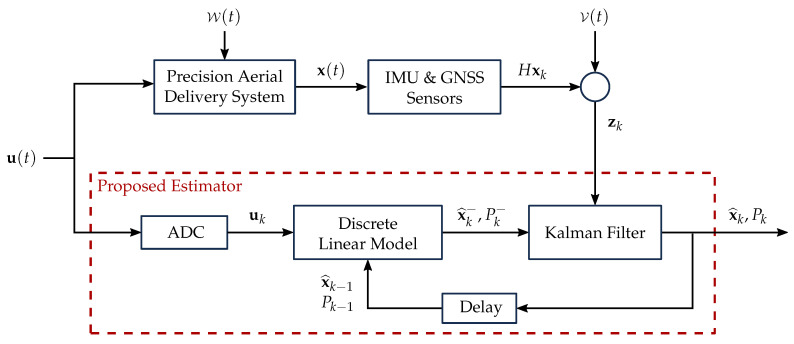
Proposed estimation scheme.

The purpose of the estimation scheme is the determination of the position and attitude of PADSs, given the availability of onboard sensors as an inertial measurement unit (IMU) and a GNSS receiver, together with a model that represents the flight dynamics of the parafoil-payload system. The estimation scheme developed and implemented in this research is presented in Figure 2.

The estimation process begins with the control inputs u(t), which are executed by the actuators of the PADS as brake deflections that modify the shape of the canopy, steering the system towards the desired landing target. The control inputs are initially processed through an analog-to-digital converter (ADC), to be incorporated into a discrete linear model representative of the flight dynamics of the system, with process noise W. The complete description of the linear models developed for this implementation is detailed in Section 3.1 and Section 3.2. The epoch-wise output states of the linear model ${\hat{x}}_{k}^{-}$ are combined with the measurements $z_{k}$ from the IMU and GNSS receiver, contaminated with noise V, through the implementation of a total state-space Kalman filter, providing the best estimate ${\hat{x}}_{k}$ of the position and attitude of the system, together with its error covariance $P_{k}$. A detailed explanation of the filter implementation is presented in Section 5.

The estimation process is performed every time a set of measurements is received from the sensors, depending on the sample rate defined for the system represented by the delay block. A comprehensive description of the sample rate and the characteristics of the sensors used is presented in Section 4. The a posteriori estimates of the filter (${\hat{x}}_{k}$, $P_{k}$) are used to predict the new a priori estimates (${\hat{x}}_{k}^{-}$, $P_{k}^{-}$), completing the recursive prediction-correction nature of the filter.

## 3. PADS Dynamic Models

To validate the accuracy of the models proposed in this research, and for comparison purposes, the 6-DOF model developed and tested by Ward et al. [29] is adopted as reference (henceforth denoted as the reference model). This model is based on the system identification of a series of flight tests, using a micro-lightweight PADS with a total mass of 2.37 kg and a canopy with a wingspan of 1.77 m, fitting the objective of this investigation. It captures the nonlinearities of the flight dynamics of the parafoil-payload system, therefore, it will serve as a reference to evaluate the performance of the linear models developed in this study.

The equations of motion of the reference model are derived from Newtonian mechanics, considering the parafoil-payload system as a rigid body, i.e., without relative motion between the parafoil and the payload. These equations are described in the body reference frame with a North-East-Down (NED) coordinate system, accounting for 6-DOF corresponding to the linear (v) and angular (ω) velocity vectors, with components (u,v,w) and (p,q,r), respectively. The dynamic equations are obtained by relating the time derivative of the linear and angular momentum to the sum of forces and moments, about the center of gravity in the body reference frame.

The forces under consideration include the aerodynamic forces that act on each element of the canopy $\left(F_{A,i}\right)$. The canopy is discretized in seven elements, allowing for brake deflections only in the outermost elements. The deflection of the left $\left(\delta_{L}\right)$ and right $\left(\delta_{R}\right)$ brakes provide steerability to the system, by changing the lift and drag coefficients of the corresponding element of the canopy. These deflections can take any value from −1 to 1 (dimensionless).

Additionally, the aerodynamic forces acting on the payload $\left(F_{A,P}\right)$, and the weight of the parafoil-payload system $\left(F_{W}\right)$ are included. Finally, apparent mass forces and moments, caused by the acceleration of the fluid through which the vehicle moves, are considered according to [30]. Since the dynamic equations are obtained in a rotating reference frame (non-inertial), fictitious forces ($F_{F}$) and moments ($M_{F}$) emerge in the equations for the change of linear and angular momentum. For a detailed explanation of the computation of each term, as well as the system identification for the parafoil-payload system, refer to the full articles [29,31].

The resulting dynamic equations of the reference model [29] (p. 591, Equations (16)–(18)), are presented in compact form in Equation (1), representing the change of the state vector as the change in linear and angular velocity in the body reference frame $\dot{x}={\left(\dot{u},\dot{v},\dot{w},\dot{p},\dot{q},\dot{r}\right)}^{T}$,

(1) $$\dot{x}={\left[GM\right]}^{-1}\left[\begin{array}{c} F_{F}+F_{W}+F_{A,P}+F_{A,i}\\ M_{F}+M_{W}+M_{A,P}+M_{A,i} \end{array}\right]$$

where the matrix GM incorporates the geometry, mass, and inertial properties of the parafoil-payload system of the reference model. While the forces and moments acting on the system are functions of the state variables in x, the aerodynamic forces and moments of the parafoil and payload, $F_{A,i}$, $F_{A,P}$, $M_{A,i}$, and $M_{A,P}$, are also functions of an external wind vector λ. For the purpose of the development and simulation of the estimation scheme proposed in this research, the three components of the external wind vector λ are assumed to be constant and equal to zero throughout the descent trajectory.

**Figure 3:**
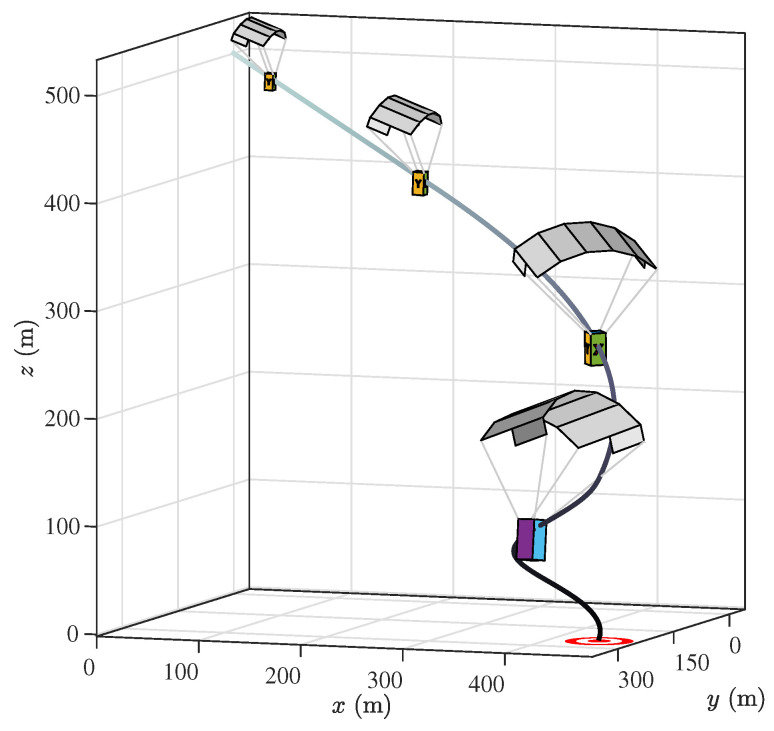
Descent trajectory under study, followed by a Precision Aerial Delivery System (PADS) navigating towards a landing target (parafoil-payload system not to scale). The trajectory corresponds to the implementation of the 6-degrees of freedom (DOF) nonlinear reference model.

By assigning initial conditions and control inputs Equation (1) can be solved, and the resulting components can be transformed to the inertial reference frame by rotating according to the Euler orientation angles. Instead of adopting the start of the descent trajectory as the origin of the inertial *z* component, this is translated to the intended point of landing to better represent visually the followed trajectory of the PADS. Figure 3 presents the trajectory under study, constructed by implementing the reference model executing the three maneuvers described in Table 1 as constant control inputs. The control inputs remain constant during the flight until the next maneuver is executed. This trajectory will serve as a reference for the analysis of the linear models presented in the following sections of this work. A video showing the reference trajectory flight is available as Supplementary Materials.

### 3.1. Linearized Dynamic Model

The linear approximation of the equations of motion is computed as the first-order Taylor series, evaluated around stable points of the states and inputs ($x_{s},u_{s}$). For the multivariable case under study $f\left(x,u\right)=f\left(u,v,w,p,q,r,\delta_{L},\delta_{R}\right)$, each of the forces and moments that the system experiences can be expressed as a power series representation in the form:

(2) $$\begin{array}{c} f\left(x,u\right)=f\left(x_{s},u_{s}\right)+\frac{\partial f}{\partial u}{|}_{x_{s}}\left(u-u_{s}\right)+\frac{\partial f}{\partial v}{|}_{x_{s}}\left(v-v_{s}\right)+\frac{\partial f}{\partial w}{|}_{x_{s}}\left(w-w_{s}\right)+\frac{\partial f}{\partial p}{|}_{x_{s}}\left(p-p_{s}\right)\\ +\frac{\partial f}{\partial q}{|}_{x_{s}}\left(q-q_{s}\right)+\frac{\partial f}{\partial r}{|}_{x_{s}}\left(r-r_{s}\right)+\frac{\partial f}{\partial \delta_{L}}{|}_{u_{s}}\left(\delta_{L}-\delta_{L_{s}}\right)+\frac{\partial f}{\partial \delta_{R}}{|}_{u_{s}}\left(\delta_{R}-\delta_{R_{s}}\right)+O \end{array}$$

where O represents higher-order terms of the Taylor series, neglected for the linearization.

Since no wind, nor other perturbations are considered, fixing the control inputs in the reference model to a given deflection brake value ($u_{s}$), result in stable states after a stabilization period of approximately 10 s, depending on the magnitude of the maneuver. Thus, for each of the three segments of constant inputs on the flight trajectory, a set of stable points is determined from the reference model as the resulting state vector. The sable points obtained for each maneuver segment, applying the deflection brakes reported in Table 1 as stable control inputs ${\delta_{L}}_{s}$ and ${\delta_{R}}_{s}$, are listed in Table 2.

Applying this linearization method to Equation (1) and grouping common terms, results in the equation of motion of the linear 6-DOF model:

(3) $$\dot{x}={\left[GM\right]}^{-1}\left[\left[\begin{array}{c} F_{s}\\ M_{s} \end{array}\right]+\left[\begin{array}{cccccc} \frac{\partial F}{\partial u} & \frac{\partial F}{\partial v} & \frac{\partial F}{\partial w} & \frac{\partial F}{\partial p} & \frac{\partial F}{\partial q} & \frac{\partial F}{\partial r}\\ \frac{\partial M}{\partial u} & \frac{\partial M}{\partial v} & \frac{\partial M}{\partial w} & \frac{\partial M}{\partial p} & \frac{\partial M}{\partial q} & \frac{\partial M}{\partial r} \end{array}\right]\left(\begin{array}{c} u-u_{s}\\ v-v_{s}\\ w-w_{s}\\ p-p_{s}\\ q-q_{s}\\ r-r_{s} \end{array}\right)+\left[\begin{array}{cc} \frac{\partial F}{\partial \delta_{L}} & \frac{\partial F}{\partial \delta_{R}}\\ \frac{\partial M}{\partial \delta_{L}} & \frac{\partial M}{\partial \delta_{R}} \end{array}\right]\left(\begin{array}{c} \delta_{L}-{\delta_{L}}_{s}\\ \delta_{R}-{\delta_{R}}_{s} \end{array}\right)\right]$$

(4) $$\dot{x}={\left[GM\right]}^{-1}\left[\left[\begin{array}{c} F_{s}\\ M_{s} \end{array}\right]+J^{x}\left(x-x_{s}\right)+J^{u}\left(u-u_{s}\right)\right]$$

where $F_{s}$ and $M_{s}$ denote the forces and moments evaluated at the stable points. In addition, $J^{x}$ and $J^{u}$ represent the corresponding Jacobian matrices. For convenience, the linearized equation of motion is presented in a state-space representation:

(5) $$\begin{array}{c} \dot{x}=Ax+Bu+\varepsilon \\ A={\left[GM\right]}^{-1}\left[J^{x}\right],\;B={\left[GM\right]}^{-1}\left[J^{u}\right],\\ \varepsilon ={\left[GM\right]}^{-1}\left[\left[\begin{array}{c} F_{s}\\ M_{s} \end{array}\right]-J^{x}\left(x_{s}\right)-J^{u}\left(u_{s}\right)\right]. \end{array}$$

Notice that matrices *A* and *B* are constant as long as the control inputs remain fixed, considerably simplifying the number of computations to be performed during flight. Similarly, all components of the vector ε are constant under the same condition, except for the components of the force produced by the weight of the vehicle ($F_{W}$), which depend on its attitude. Matrices *A* and *B*, as well as vector ε, evaluated at the stable points for each maneuver segment, are given in Appendix A.

The complete description of the position and attitude of the PADS in the inertial reference frame, as well as the linear and angular velocity in the body reference frame, can be expressed in a concise form by combining the kinematic and dynamic equations of motion in a single state-space representation:

(6) $$\left[\begin{array}{c} \dot{p}\\ \dot{\Omega }\\ \dot{v}\\ \dot{\omega } \end{array}\right]=\left[\begin{array}{cccc} 0 & 0 & T_{BI} & 0\\ 0 & 0 & 0 & S_{BI}\\ 0 & 0 & A_{11} & A_{12}\\ 0 & 0 & A_{21} & A_{22} \end{array}\right]\left[\begin{array}{c} p\\ \Omega \\ v\\ \omega \end{array}\right]+\left[\begin{array}{cc} 0 & 0\\ 0 & 0\\ B_{11} & B_{11}\\ B_{21} & B_{22} \end{array}\right]\left[\begin{array}{c} \delta_{L}\\ \delta_{R} \end{array}\right]+\left[\begin{array}{c} 0\\ 0\\ \varepsilon_{1}\\ \varepsilon_{2} \end{array}\right]$$

where matrices *A* and *B*, as well as the vector ε are distributed in components $A_{ij}$, $B_{ij}$, and $\varepsilon_{i}$ respectively, to accommodate an adequate state-space representation. Additionally, $T_{BI}$ and $S_{BI}$ are the rotation matrices that transform the linear and angular velocity components from the body to the inertial reference frame:

(7) $$T_{BI}=\left[\begin{array}{ccc} c_{\theta }c_{\psi } & s_{\phi }s_{\theta }c_{\psi }-c_{\phi }s_{\psi } & c_{\phi }s_{\theta }c_{\psi }+s_{\phi }s_{\psi }\\ c_{\theta }s_{\psi } & s_{\phi }s_{\theta }s_{\psi }+c_{\phi }c_{\psi } & c_{\phi }s_{\theta }s_{\psi }-s_{\phi }c_{\psi }\\ -s_{\theta } & s_{\phi }c_{\theta } & c_{\phi }c_{\theta } \end{array}\right]$$

(8) $$S_{BI}=\left[\begin{array}{ccc} 1 & s_{\phi }t_{\theta } & c_{\phi }t_{\theta }\\ 0 & c_{\phi } & -s_{\phi }\\ 0 & s_{\phi }/c_{\theta } & c_{\phi }/c_{\theta } \end{array}\right]$$

where the shorthand notation $c_{*}$, $s_{*}$, and $t_{*}$ denotes cos(∗), sin(∗), and tan(∗), respectively. Rewriting Equation (6) leads to a compact equation, where the superscript Li is used to indicate that an element corresponds to the linearized model:

(9) $${\dot{x}}^{Li}=A^{Li}x^{Li}+B^{Li}u^{Li}+\varepsilon^{Li}.$$

While Equation (9) represents a time-continuous model, information from the onboard sensors arrive at discrete times. Solving the differential equation for the state vector using Euler’s method, with time step Δt, a time-discrete model is obtained as

(10) $$\begin{array}{c} x_{k}^{Li}=\left(A^{Li}\Delta t+I\right)x_{k-1}^{Li}+B^{Li}\Delta tu_{k-1}^{Li}+\varepsilon_{k-1}^{Li}\Delta t\\ x_{k}^{Li}=F^{Li}x_{k-1}^{Li}+G^{Li}u_{k-1}^{Li}+\epsilon_{k-1}^{Li}. \end{array}$$

where *I* represents the identity matrix. It is relevant to observe that Equation (10) describes 12 components in total, three for each vector: the position vector $p={\left(x,y,z\right)}^{T}$ and the angular position vector $\Omega ={\left(\phi ,\theta ,\psi \right)}^{T}$, expressed in the inertial reference frame, with the latter corresponding to the Euler orientation angles (roll, pitch, yaw); and the linear velocity vector $v={\left(u,v,w\right)}^{T}$ and angular velocity vector $\omega ={\left(p,q,r\right)}^{T}$, expressed in the body reference frame.

### 3.2. Double Integrator Dynamic Model

The development of an alternative linear model is achieved by exploiting the properties of the flight dynamics of PADSs. Since these are vehicles navigating in an underactuated controlled descent flight, typically without propulsion, and subject only to variations in the wind profiles, the flight dynamics are normally smooth, i.e., without sudden changes in the state variables of the parafoil-payload system, except for the voluntary control inputs exerted on the vehicle. Taking this into consideration, a double integrator model is proposed, based on the assumption that between consecutive measurements of the sensors acquiring information regarding the position and attitude of the vehicle, the linear $\left(\ddot{p}\right)$ and angular $\left(\ddot{\Omega }\right)$ accelerations remain constant for Δt seconds. Integrating twice these accelerations with respect to time leads to the following equations of motion for the position and attitude, in the inertial reference frame:

(11) $$p_{k}=p_{k-1}+{\dot{p}}_{k-1}\Delta t+{\ddot{p}}_{k-1}\Delta t^{2}/2$$

(12) $$\Omega_{k}=\Omega_{k-1}+{\dot{\Omega }}_{k-1}\Delta t+{\ddot{\Omega }}_{k-1}\Delta t^{2}/2$$

which can be expressed in compact form as

(13) $${\left[\begin{array}{c} p\\ \dot{p}\\ \Omega \\ \dot{\Omega } \end{array}\right]}_{k}=\left[\begin{array}{cccc} 1 & \Delta t & 0 & 0\\ 0 & 1 & 0 & 0\\ 0 & 0 & 1 & \Delta t\\ 0 & 0 & 0 & 1 \end{array}\right]{\left[\begin{array}{c} p\\ \dot{p}\\ \Omega \\ \dot{\Omega } \end{array}\right]}_{k-1}+\left[\begin{array}{cc} \frac{\Delta t^{2}}{2} & 0\\ \Delta t & 0\\ 0 & \frac{\Delta t^{2}}{2}\\ 0 & \Delta t \end{array}\right]{\left[\begin{array}{c} \ddot{p}\\ \ddot{\Omega } \end{array}\right]}_{k-1}$$

(14) $$x_{k}^{D}=F^{D}x_{k-1}^{D}+G^{D}u_{k-1}^{D}$$

where the superscript *D* is used to indicate that an element corresponds to the double integrator model. Notice that Equation (14) also describes 12 components in total, corresponding to the position, linear velocity, angular position, and angular velocity vectors, in the inertial reference frame. The position and angular position vectors can be directly compared with the analogous components of the nonlinear and linearized models. Contrarily, the linear and angular velocity vectors obtained from the double integrator model cannot be directly compared with the similar components from the nonlinear or linearized models, since they are expressed in different reference frames. To be able to compare them, the linear and angular velocity vectors obtained from the double integrator model are transformed to the body reference frame with the rotation matrices $T_{BI}^{-1}$ and $S_{BI}^{-1}$, respectively.

## 4. Sensors Characterization

The hardware under consideration must comply with the requirements of a lightweight mission, where the allocated volume and weight for the low-cost sensors are very limited. Since this estimation scheme is intended to be used in low-cost applications.

To evaluate the performance of the estimation scheme, simulated measurements are generated at 5 Hz sample rate, based on the state variables of the reference model and the statistical characteristics of the sensors acquiring this information in practice. This characterization is obtained through experimentation with real flight data from similar vehicles. Specifically, measurements from a GNSS receiver, a magnetometer, a gyroscope, and an accelerometer, are simulated by corrupting the position, angular position, angular velocity, and linear acceleration, of the reference model with white Gaussian noise, according to the statistical parameters reported in Table 3.

The obtained deviation values from experimentation are similar in comparison to the noise and bias values reported in similar experiments. For example, Slegers and Yakimenko [32] present a bias in global positioning system (GPS) measurements of 2 m, plus noise of 0.5 m; 2 deg bias and 1 deg noise for the angular position; and finally, 1 deg/s bias and 1 deg/s noise for the angular velocity. In their study, they also draw upon a linearization process of the flight dynamics with a sample rate of 2 Hz, assuming constant aerodynamic velocity. Cacan et al. [23] report 4 Hz sample rate for their guidance, navigation, and control algorithm, of a micro-lightweight PADS. Ward et al. [31] report noise with a standard deviation of 2 m in positioning measurements, and a standard deviation of 10 deg and 2 deg for the heading measurement bias and noise respectively, using 4 Hz sampling rate.

The sampling rate of 5 Hz for our investigation was chosen according to the processing capabilities of typical low-end microprocessors and sensors. For example, low-cost GNSS receivers as the u-blox NEO-6M or NEO-M8N can provide navigation information up to 1 Hz to 5 Hz, limiting the measurement availability [33,34].

Typical performance of processing units for this lightweight and low-cost application range from a microprocessor with 8-bit, 20 MHz capabilities, as the ATmega328 chip [35]; 32-bit, 216 MHz as the STM32F765 chip [36]; up to 64-bit, 1.5 GHz as the BCM2711 chip [37].

## 5. Kalman Filter Estimation Algorithm

Typically, PADSs are equipped with a GNSS receiver and an IMU, that provide information at discrete times about the position and attitude of the vehicle during its flight. The objective of any guidance, navigation, and control scheme is to process the incoming information from the sensors, in order to estimate the state variables of the system, to plan the most suitable trajectory towards the landing target while managing the energy budget. Given a linear discrete-time model for the state vector x and a measurement vector z:

(15) $$x_{k}=F_{k-1}x_{k-1}+G_{k-1}u_{k-1}+W_{k-1}$$

(16) $$z_{k}=Hx_{k}+V_{k}$$

with process noise W and measurement noise V, both assumed to be white, uncorrelated, zero-mean, and normally-distributed, the Kalman filter provides the best estimate of the states ${\hat{x}}_{k}$. In the following, the implementation of a model-based Kalman filter is described using a total state-space formulation [38].

Initially, an a priori state estimate ${\hat{x}}_{k}^{-}$ and error covariance estimate $P_{k}^{-}$ are obtained by propagating the a posteriori estimate from epoch k−1 to *k*:

(17) $${\hat{x}}_{k}^{-}=F_{k-1}{\hat{x}}_{k-1}+G_{k-1}u_{k-1}$$

(18) $$P_{k}^{-}=F_{k-1}P_{k-1}F_{k-1}^{T}+Q_{k-1}$$

where *Q* represent the process noise covariance matrix, estimated using the autocorrelation function $Q=G\sigma^{2}G^{T}$, with $\sigma_{Li}^{2}=800$ and $\sigma_{D}^{2}=10$ for each model, determined by tuning the filter during its implementation. Note that Equation (17) has the same form as the state vector obtained in Equation (10) for the linearized model, as well as in Equation (14) for the double integrator model, developed in the previous sections. Next, the a posteriori state estimate ${\hat{x}}_{k}$ is computed as a linear combination of the a priori estimate ${\hat{x}}_{k}^{-}$, and the difference between the measurement vector $z_{k}$ and the prediction $H{\hat{x}}_{k}^{-}$, weighted by the Kalman gain $K_{k}$, designed to minimize the a posteriori error covariance $P_{k}$:

(19) $$K_{k}=P_{k}^{-}H^{T}{\left(HP_{k}^{-}H^{T}+R_{k}\right)}^{-1}$$

(20) $${\hat{x}}_{k}={\hat{x}}_{k}^{-}+K_{k}\left(z_{k}-H{\hat{x}}_{k}^{-}\right)$$

(21) $$P_{k}=\left(I-K_{k}H\right)P_{k}^{-}{\left(I-K_{k}H\right)}^{T}+K_{k}R_{k}K_{k}^{T}$$

where *H* denotes the observation matrix, *R* represents the measurement noise covariance matrix, and *I* corresponds to the identity matrix. The simulated measurements are incorporated into the Kalman filter as the measurement vector z, while the squared of the standard deviations of the characterized sensors are used as the elements of the diagonal measurement noise covariance matrix *R*. The observation matrix *H* communicates the availability of the measurements, relating them with the state vector.

The implementation of the filter using the linearized model is realized by executing the constant brake deflections described in Table 1 as control inputs, fusing the simulated measurements obtained by the sensors. On the other hand, the double integrator model requires linear and angular accelerations as control inputs, which typically can be deduced from measurements employing onboard sensors. These accelerations are also simulated based on the reference model, by transforming the linear and angular acceleration from the body reference frame to the inertial reference frame, and adding white Gaussian noise with standard deviations of 0.12 m/s${}^{2}$ and 2 deg/s${}^{2}$ respectively, according to experimental data.

## 6. Simulation Results

Two separate implementations of the described discrete Kalman filter scheme were performed: one using the linearized model and brake deflections as control inputs, and another using the double integrator model and the simulated accelerations as control inputs. Both implementations were performed using the same initial conditions and simulated measurements.

**Figure 4:**
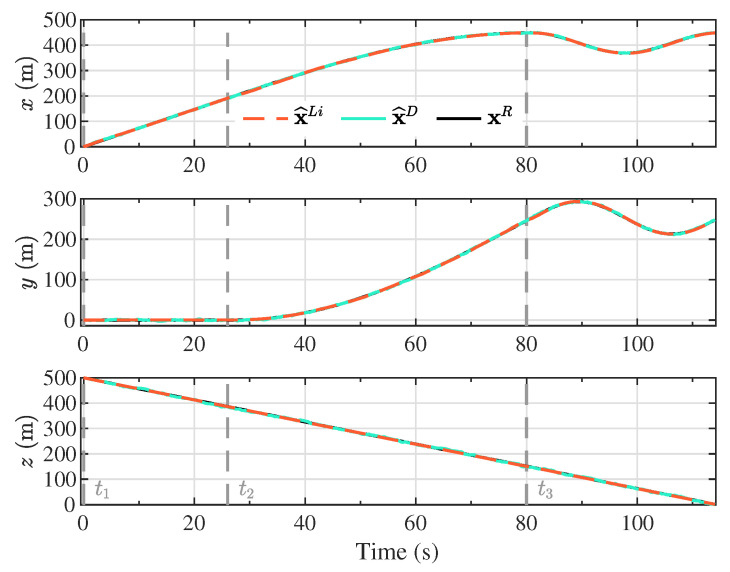
Kalman filter inertial position estimates based on the proposed linearized model $\left({\hat{x}}^{Li}\right)$ and the double integrator model $\left({\hat{x}}^{D}\right)$, together with the components of the inertial position vector from the reference model $\left(x^{R}\right)$.

**Figure 5:**
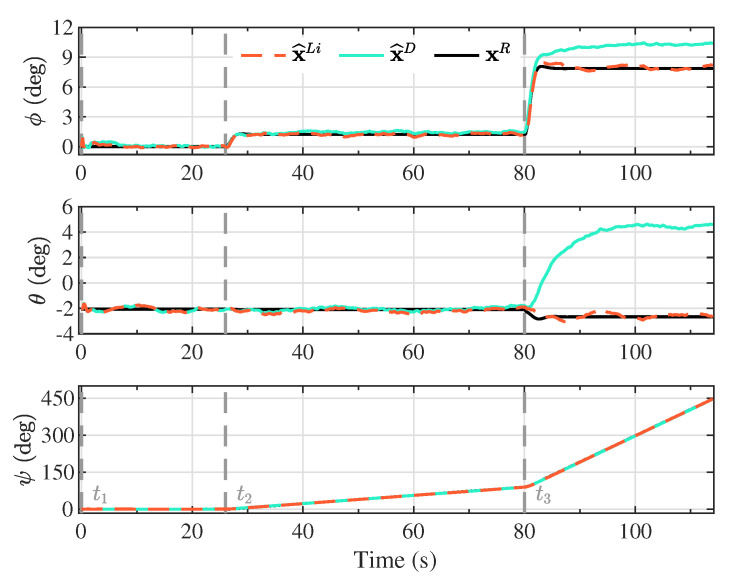
Kalman filter angular position estimates based on the proposed linearized model $\left({\hat{x}}^{Li}\right)$ and the double integrator model $\left({\hat{x}}^{D}\right)$, together with the components of the angular position vector from the reference model $\left(x^{R}\right)$. These components are expressed in the inertial reference frame, corresponding to the Euler orientation angles roll, pitch, and yaw, respectively.

**Figure 6:**
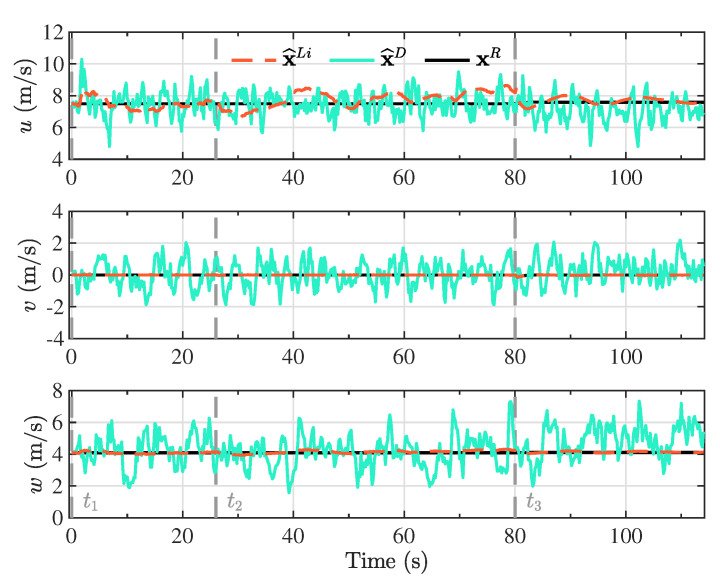
Kalman filter linear velocity estimates based on the proposed linearized model $\left({\hat{x}}^{Li}\right)$ and the double integrator model $\left({\hat{x}}^{D}\right)$, together with the components of the linear velocity vector from the reference model $\left(x^{R}\right)$. These components are expressed in the body reference frame.

**Figure 7:**
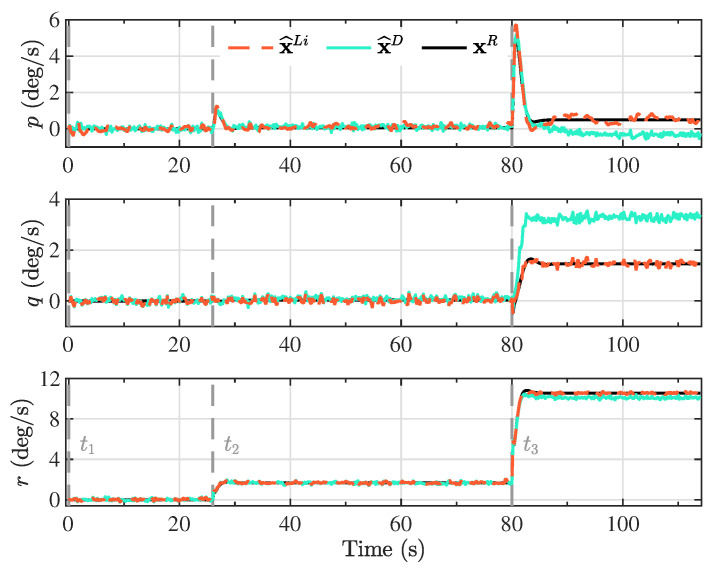
Kalman filter angular velocity estimates based on the proposed linearized model $\left({\hat{x}}^{Li}\right)$ and the double integrator model $\left({\hat{x}}^{D}\right)$, together with the components of the angular velocity vector from the reference model $\left(x^{R}\right)$. These components are expressed in the body reference frame.

The Kalman filter estimates based on the proposed linearized model (${\hat{x}}^{Li}$) and the double integrator model $\left({\hat{x}}^{D}\right)$ are presented in Figure 4, Figure 5, Figure 6 and Figure 7, together with the reference model $\left(x^{R}\right)$ for comparison purposes. The different maneuver segments are presented in all figures with vertical dashed lines at the time of execution, with the maneuver identifier displayed at the bottom to facilitate the interpretation.

From Figure 4, it can be observed that the Kalman filtering scheme based on each of the proposed models was capable of reproducing the components of the inertial position of the vehicle, for any of the flight scenarios corresponding to the maneuver segments. Due to the smooth evolution of each of the components of the inertial position, both the linearized model and the double integrator model were suitable for emulating the behavior of the position of the nonlinear reference model. This mild progression of the inertial position of the PADS closely represented a real flight scenario. From the beginning of the descent trajectory, after the full inflation of the canopy and stabilization of the gliding flight, the only external perturbation experienced by the parafoil-payload system originated from the wind. While strong wind profiles could substantially affect the flight dynamics of small vehicles, a fuselage designed to reduce drag could be employed to mitigate this effect. Additionally, the heavier the payload, the less prone it was to suffer abrupt changes in position due to its inertia.

The components of the linear velocity, presented in Figure 6, demonstrate that the lateral component of the velocity (*v*) was virtually zero along the complete descent trajectory. This reflects the fact that there were no perturbations that modify the lateral movement of the vehicle aside from the control inputs, and that the flight was dominated by its longitudinal dynamics. In the three linear velocity components, it was distinctly recognizable that the estimations based on the double integrator model strongly varied around the reference model, in comparison with the estimates from the linearized model. While the bounded variation confirmed that the Kalman filter estimation scheme converged towards the reference model, it was an indication that the Kalman gain was favoring the measurements instead of the model.

Note that along the descent trajectory, the deflection brakes applied on each maneuver were held constant until the next maneuver was reached, or the vehicle landed. Furthermore, the maneuvers were applied instantaneously, as step functions. This means that the delayed response of the actuators, and the elasticity of the lines used to apply the deflection brakes were not taken into account, which would modify the transient response of the state variables. Despite the condition analyzed in this investigation where the brake deflections were applied infinitely fast, the estimation process based on the proposed linearized model was able to follow the dynamics of the reference model, suggesting that for smoother executions of the maneuvers, the performance only could improve. In real applications, the maneuvers cannot be applied instantaneously, but gradually, leading to a smoother response on the state variables. The consequence of this abrupt change in the maneuver segments can be observed, for example, in the angular velocity components (p,q,r) in Figure 7, especially when the last maneuver $\left(t_{3}\right)$ is applied.

While Figure 4, Figure 5, Figure 6 and Figure 7 provide valuable information about the 12 components of the state vector estimated by virtue of the proposed Kalman filter scheme, it is difficult to appreciate the shape and magnitude of the error with respect to the reference model. The error was computed as the difference between the reference model $x^{R}$ and the Kalman filter state estimates based on the linearized model ${\hat{x}}^{Li}$ and the double integrator model ${\hat{x}}^{D}$, respectively. This error is denoted as ${\bar{x}}^{Li}$ and ${\bar{x}}^{D}$ for each implementation.

**Figure 8:**
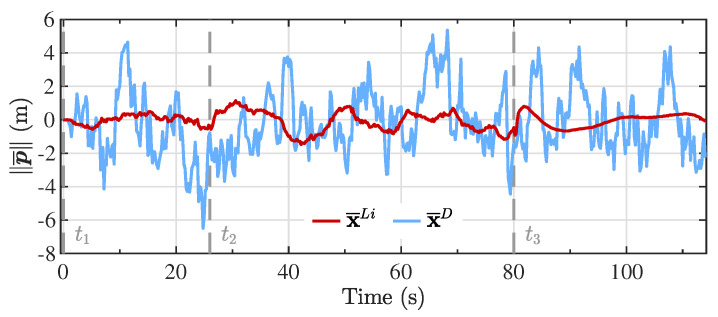
Error in the magnitude of the inertial position vector between the reference model and the Kalman filter estimates from the linearized model $\left({\bar{x}}^{Li}\right)$ and the double integrator model $\left({\bar{x}}^{D}\right)$.

To obtain a better understanding of how closely the estimation scheme followed the reference model for the inertial position, the error in the magnitude of the position vector obtained with the reference model and the magnitude of the Kalman filter position vector estimates are presented in Figure 8, denoted as $∥\bar{p}∥$. It can be appreciated that both models were capable of representing the tridimensional position of the vehicle during the different flight segments since in general, the error oscillated around zero. In contrast, the filter estimates based on the linearized model presented a smoother variation, with an error magnitude approximately two times smaller.

**Figure 9:**
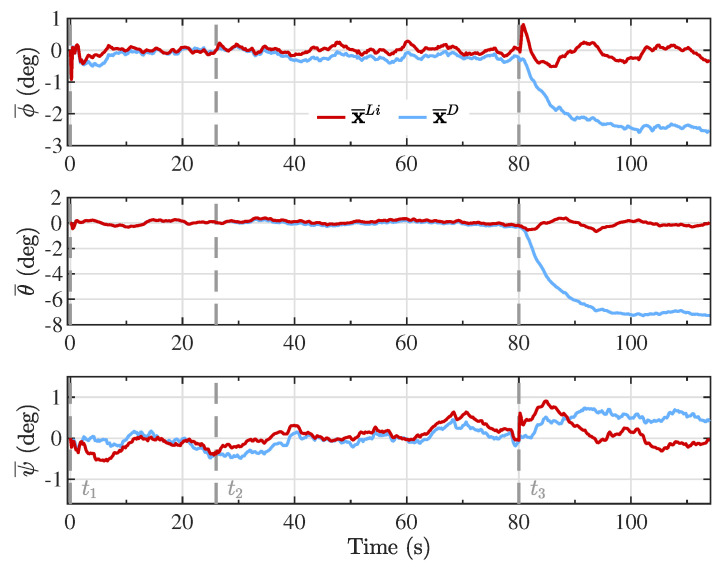
Error in the inertial angular position components between the reference model and the Kalman filter estimates from the linearized model (${\bar{x}}^{Li}$) and the double integrator model (${\bar{x}}^{D}$).

**Figure 10:**
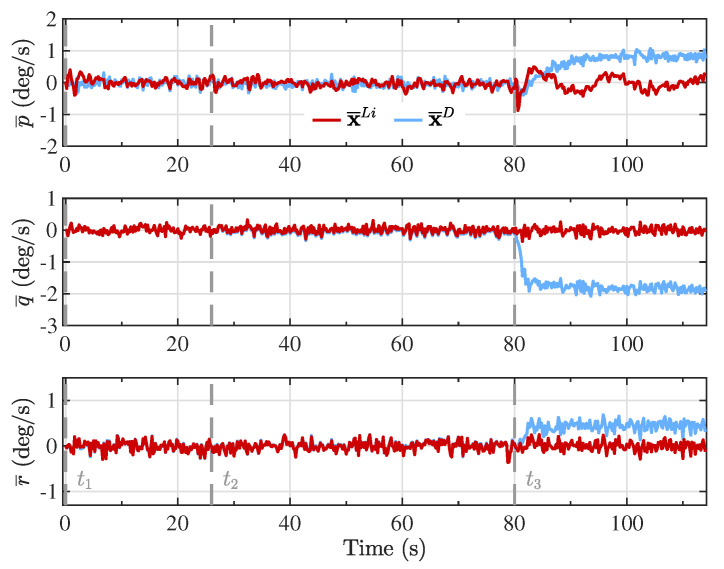
Error in the angular velocity components, expressed in the body reference frame, between the reference model and the Kalman filter estimates from the linearized model (${\bar{x}}^{Li}$) and the double integrator model (${\bar{x}}^{D}$).

To evaluate the performance in estimating the attitude of the PADS, Figure 9 presents the error between the Euler angles of the reference model and the Kalman filter estimates using each of the proposed models. Notice that during the first and second maneuver segments ($t_{1}$ and $t_{2}$), corresponding to a straight flight and a wide turn, the two models captured the same behavior as the reference model. Nevertheless, the double integrator model presented a larger and consistent error for roll (ϕ) and pitch (θ) angles, and a smaller but still noticeable constant error for the yaw angle (ψ), after the third maneuver was applied ($t_{3}$), where the flight trajectory with a narrower turn was followed. This is a consequence of the higher rate of change in the angular position that the vehicle experienced during the last flight segment, where a −0.4 right brake deflection was being applied. The double integrator model relied on the assumption that the parafoil-payload system experienced epoch-wise constant inertial acceleration during the integration period. Consequently, the higher the body-fixed angular velocity, the less this assumption was fulfilled, as can be verified from the error in the angular velocity components presented in Figure 10.

The achievable turn rate for any PADS has a practical limit, since a steep turn rate could induce spiral divergence. For a vehicle with similar characteristics as the simulated in this study, Ward et al. [39,40] report maximum turn rates from 15 deg/s up to 25 deg/s, depending on the flight mode and control scheme. For the case of larger parafoil-payload vehicles, Lingard [11] reports a maximum constant turn rate of 11.5 deg/s for a canopy with 30 m of wingspan.

## 7. Discussion

It is relevant to highlight some properties and prerequisites for the operation and deduction of the linearized model and the double integrator model. The linearized model requires the calculation of the Jacobian matrices, increasing the complexity of its deduction depending on the forces and moments representing the interaction between the vehicle and its surroundings. This requires the characterization of the parafoil-payload system, limiting the flexibility of this model to be applied in any other vehicle with different properties. Moreover, the stable points need to be computed prior to the descent trajectory. While this demands additional effort, it is important to note that this task is performed on the ground, where typically more computational resources are available, without increasing the real-time computational burden during the flight. Since the flight conditions variate from flight to flight, it is required to calculate a set of stable points assuming the planned maneuvers to reach the target. If the computed stable points differ greatly from the actual conditions during flight, the performance would deteriorate.

In contrast, the double integrator model does not depend on the physical properties of the parafoil-payload system, simplifying the implementation of this model on vehicles with different characteristics. As a consequence, this model does not directly relate the brake deflections to the state variables, making it not suitable for a straightforward application of a control scheme for guidance. While the implementation of this model is much simpler than the linearized model, without requiring the computation of the stable points or other parameters before the flight, it heavily depends on the sample rate and quality of the measurements provided by the sensors.

The selected 5 Hz sample rate for the measurements is a conservative choice that complies with the capacities of any relatively modern low-cost processing unit and sensors for real-time applications. The higher the capabilities of the sensors and the processing unit, the larger the achievable sample rate during flight. This might be desirable when fast maneuvers are required or strong wind profiles modify the trajectory of the vehicle, although this implies higher costs associated with instrumentation, energy storage, and a heavier payload, sometimes not feasible for micro-lightweight PADSs.

In the case that exogenous forces are present during the flight as a consequence of strong winds, which are not incorporated into the flight dynamic models as the external wind vector λ, it is expected that the predictions provided by the models would deteriorate. Another source of perturbations could be the effect of mismodeled interactions in the parafoil-payload system, due to the relative motion of its components. Nevertheless, the overall performance of the proposed estimation scheme would not be necessarily deteriorated proportionally to the magnitude of the perturbations, since the Kalman gain would favor the measurements from the sensors or the predictions of the dynamic models, attempting to compensate for these effects by minimizing the a posteriori error covariance.

## 8. Conclusions

Throughout this research article, two models that represent the flight dynamics of micro-lightweight PADSs were developed: a linearized version of a 6-DOF nonlinear model and a double integrator model. Additionally, simulated measurements representative of sensors onboard the vehicle were fused by implementing a Kalman filter algorithm based on the developed models, to estimate the position and attitude of the parafoil-payload system.

The simulation results demonstrate that both models capture the flight dynamics of micro-lightweight PADSs when incorporated into a Kalman filtering scheme for smooth flights. While the double integrator model excels in simplicity, and it is independent of the physical properties of the parafoil-payload system, it falls behind in precision capturing the flight dynamics, especially when the vehicle is subject to intense accelerations. On the other hand, the linearized model is capable of representing the flight dynamics of the vehicle more accurately and preserves precision even during narrow maneuvers, but depends on the determination of stable points close to the operation point of the vehicle.

Further investigation is required on the inclusion of the proposed estimation scheme into a global closed-loop control strategy for the vehicle. In particular, tracking of constant waypoints is usually a control objective for this type of system. The utilization of the obtained linearized model could be a solid starting point for the development of a model-based robust controller. The design of a suitable control strategy, that includes the proposed estimation scheme, and its stability analysis is left as future work.

## Figures and Tables

**Table 1:** Control maneuver segments.

Maneuver Id.	Start (s)	$\delta_{L}$	$\delta_{R}$
$t_{1}$	0	0	0
$t_{2}$	26	0.1	0
$t_{3}$	80	0	−0.4

**Table 2:** Stable points.

State Variable	Maneuver Segment		
$x_{s}$	$t_{1}$	$t_{2}$	$t_{3}$
$u_{s}$ (m/s)	7.49	7.49	7.59
$v_{s}$ (m/s)	0	0	−0.01
$w_{s}$ (m/s)	4.09	4.09	4.12
$p_{s}$ (deg/s)	0	0.06	0.50
$q_{s}$ (deg/s)	0	0.04	1.46
$r_{s}$ (deg/s)	0	1.67	10.55

**Table 3:** Sensors statistical parameters.

Sensor	Measurement Standard Deviation
GNSS (xy)	1.8 m
GNSS (z)	5 m
Magnetometer	0.5 deg
Gyroscope	0.1 deg/s
Accelerometer	0.12m/s${}^{2}$

## Supplementary Materials

The following are available online at https://www.mdpi.com/1424-8220/20/18/5227/s1, Video S1: reference trajectory. All the code developed for this research is openly available at doi: 10.5281/zenodo.3978828.

## Abbreviations

The following abbreviations are used in this manuscript:

ADC	Analog-to-Digital Converter
AGAS	Affordable Guided Airdrop System
ALEX	Small Autonomous Parafoil Landing Experiment
CEP	Circular Error Probability
COVID-19	Coronavirus Disease 2019
DOF	Degrees of Freedom
GNSS	Global Navigation Satellite System
GPS	Global Positioning System
IMU	Inertial Measurement Unit
JPADS	Joint Precision Airdrop System
LiDAR	Light Detection and Ranging
NED	North-East-Down
PADS	Precision Aerial Delivery System
UAV	Unmanned Aerial Vehicles

## Appendix A

In the following, the components of matrices *A* and *B*, and vector ε, are presented, corresponding to each of the maneuver segments for the linearized model. These values are obtained by evaluating the forces, moments, and Jacobian matrices at the stable points reported in Table 2 for the state variables $x_{s}$, and the brake deflections reported in Table 1 as control inputs $u_{s}$. Consequently, their values remain constant throughout each of the control segments. The only elements that are not constant are the components of the force vector caused by the weight of the parafoil-payload system ($F_{W}$), which depend on the instant attitude and need to be calculated epoch-wise. Recalling:

$$\begin{array}{c} A={\left[GM\right]}^{-1}\left[J^{x}\right]\;\\ B={\left[GM\right]}^{-1}\left[J^{u}\right]\;\\ \varepsilon ={\left[GM\right]}^{-1}\left[\left[\begin{array}{c} F_{s}\\ M_{s} \end{array}\right]-J^{x}\left(x_{s}\right)-J^{u}\left(u_{s}\right)\right]. \end{array}$$

The matrix GM incorporates information regarding the geometry, mass, and inertia of the parafoil-payload system [29] (p. 591, Equation (16)). These attributes are fixed, since the vehicle is considered a rigid body, and therefore this matrix remains constant throughout the descent trajectory:

$${\left[GM\right]}^{-1}=\left[\begin{array}{rrrrrr} 0.42 & 0 & 0 & 0 & 0.02 & 0\\ 0 & 0.41 & 0 & -0.1 & 0 & 0.05\\ 0 & 0 & 0.33 & 0 & 0 & 0\\ 0 & -0.1 & 0 & 1.76 & 0 & -0.89\\ 0.02 & 0 & 0 & 0 & 2.28 & 0\\ 0 & 0.05 & 0 & -0.89 & 0 & 17.43 \end{array}\right]$$

Maneuver segment 1:

$$A=\left[\begin{array}{rrrrrr} -0.97 & 0 & 1.61 & 0 & -2.99 & 0\\ 0 & -1.09 & 0 & 3.19 & 0 & -7.14\\ 0.54 & 0 & -4.76 & 0 & 5.24 & 0\\ 0 & -4.23 & 0 & -5.18 & 0 & 2.34\\ 6.11 & 0 & -11.2 & 0 & -7.32 & 0\\ 0 & 8.74 & 0 & 7.26 & 0 & -6.68 \end{array}\right]$$

$$B=\left[\begin{array}{rr} -0.047 & -0.047\\ 0.01 & -0.01\\ -0.021 & -0.021\\ -0.174 & 0.174\\ 0.193 & 0.193\\ 1.683 & -1.683 \end{array}\right]$$

$$\varepsilon ={\left[GM\right]}^{-1}\left[\begin{array}{c} {F_{W}}_{x}\\ {F_{W}}_{y}\\ {F_{W}}_{z}\\ 0\\ 0\\ 0 \end{array}\right]+\left[\begin{array}{r} 0.35\\ 0\\ 7.721\\ 0\\ 0.018\\ 0 \end{array}\right]$$

Maneuver segment 2:

$$A=\left[\begin{array}{rrrrrr} -0.97 & 0.03 & 1.61 & 0 & -2.99 & 0\\ -0.03 & -1.1 & 0 & 3.19 & 0 & -7.14\\ 0.54 & 0 & -4.76 & 0 & 5.23 & 0\\ 0.01 & -4.24 & -0.01 & -5.19 & 0.01 & 2.35\\ 6.11 & -0.01 & -11.19 & -0.04 & -7.32 & 0\\ 0.01 & 8.75 & 0.02 & 7.28 & 0 & -6.72 \end{array}\right]$$

$$B=\left[\begin{array}{rr} -0.052 & -0.048\\ 0.011 & -0.01\\ -0.023 & -0.021\\ -0.19 & 0.175\\ 0.21 & 0.194\\ 1.838 & -1.693 \end{array}\right]$$

$$\varepsilon ={\left[GM\right]}^{-1}\left[\begin{array}{c} {F_{W}}_{x}\\ {F_{W}}_{y}\\ {F_{W}}_{z}\\ 0\\ 0\\ 0 \end{array}\right]+\left[\begin{array}{r} 0.359\\ 0.201\\ 7.721\\ -0.031\\ -0.003\\ -0.158 \end{array}\right]$$

Maneuver segment 3:

$$A=\left[\begin{array}{rrrrrr} -0.98 & 0.21 & 1.6 & 0.02 & -3.02 & -0.02\\ -0.17 & -1.1 & 0 & 3.21 & -0.01 & -7.24\\ 0.57 & 0 & -4.79 & 0.01 & 5.31 & 0\\ 0.06 & -4.24 & -0.06 & -5.18 & 0.08 & 2.36\\ 6.13 & -0.09 & -11.27 & -0.25 & -7.35 & 0\\ 0.05 & 8.72 & 0.13 & 7.19 & 0.02 & -6.51 \end{array}\right]$$

$$B=\left[\begin{array}{rr} -0.047 & -0.13\\ 0.01 & -0.026\\ -0.021 & -0.057\\ -0.17 & 0.48\\ 0.188 & 0.532\\ 1.645 & -4.65 \end{array}\right]$$

$$\varepsilon ={\left[GM\right]}^{-1}\left[\begin{array}{c} {F_{W}}_{x}\\ {F_{W}}_{y}\\ {F_{W}}_{z}\\ 0\\ 0\\ 0 \end{array}\right]+\left[\begin{array}{r} 0.4\\ 1.279\\ 7.622\\ -0.128\\ 0.235\\ -1.698 \end{array}\right]$$
